# Undirected (Solitary) Birdsong in Female and Male Blue-Capped Cordon-Bleus (*Uraeginthus cyanocephalus*) and Its Endocrine Correlates

**DOI:** 10.1371/journal.pone.0026485

**Published:** 2011-10-19

**Authors:** Nicole Geberzahn, Manfred Gahr

**Affiliations:** Department of Behavioural Neurobiology, Max Planck Institute for Ornithology, Seewiesen, Germany; University of Sussex, United Kingdom

## Abstract

**Background:**

Birdsong is a popular model system in research areas such as vocal communication, neuroethology or neuroendocrinology of behaviour. As most research has been conducted on species with male-only song production, the hormone-dependency of male song is well established. However, female singing and its mechanisms are poorly understood.

**Methodology/Principal Findings:**

We characterised the song and its endocrine correlates of blue-capped cordon-bleus (*Uraeginthus cyanocephalus*), a species in which both sexes sing. Like other estrildids, they produce directed song during courtship and undirected (or solitary) song in isolation, i.e. when the mate is not visible or absent. We compare solitary song of blue-capped cordon-bleus to published descriptions of the song of its relative, the zebra finch (*Taeniopygia guttata*). Solitary song of cordon-bleus shared some overall song features with that of zebra finches but differed in spectro-temporal song features, sequential stereotypy and sequential organisation. The song of cordon-bleus was dimorphic with respect to the larger size of syllable repertoires, the higher song duration and the lower variability of pitch goodness (measuring the pureness of harmonic sounds) in males. However, in both sexes the overall plasma testosterone concentrations were low (ca. 300 pg/ml) and did not correlate with the sexually dimorphic song motor pattern. Despite such low concentrations, the increase in the rate of solitary song coincided with an increase in the level of testosterone. Furthermore, the latency to start singing after the separation from the mate was related to hormone levels.

**Conclusions/Significance:**

Our findings suggest that the occurrence of solitary song but not its motor pattern might be under the control of testosterone in female and male cordon-bleus.

## Introduction

Birdsong has been studied mainly in species that breed in the temperate zone, and in most of them, only male birds sing. However, the majority of bird species breed in the tropics [1], and females of many of the tropical species sing as well [2]. The neglected issue of female song production started to attract more attention recently, (e.g. [3]–[13]), but the physiology (e.g. endocrine control) of female song production as well as of sex-specific differences in singing behaviour are still poorly understood. We have recently established a breeding colony of blue-capped cordon-bleus (*Uraeginthus cyanocephalus*), to study song production, song development, song learning and endocrine effects on song in this species both in females and males in the laboratory. Blue-capped cordon-bleus are members of the same songbird family (the estrildid finches) as the widely studied zebra finch (*Taeniopygia guttata*) but differ from the latter in that the females regularly sing.

Blue-capped cordon-bleus are small, moderately gregarious, sexually dimorphic with respect to plumage colouration and socially monogamous estrildid finches occurring in semi-arid and arid regions of East Africa. They are opportunistic breeders and only defend an area around the nest but no territories [14]. Both sexes sing and their song plays an important role in inter-sexual communication, i.e., song is an advertising signal addressed to mates, whereas it has no function in territorial defence [15]. Like other estrildids, blue-capped cordon-bleus mainly produce song in two different social contexts: (1) during and as part of the courtship display (directed or courtship song) and (2) apart from the courtship context (undirected song), particularly when the mate is not visible or absent.

Directed and undirected song has been intensively studied in the zebra finch, a species in which only males sing. Whilst zebra finches use the same song type in both contexts there are subtle differences in the way that song type is performed: song performance is less variable in directed than in undirected song, during directed singing song units are repeated more often, song is performed faster and has a more ‘rigid’ syntax [16]. Furthermore, syllables are produced with significantly higher spectral stereotypy [17]. Such behavioural differences are paralleled by differences in the neuronal activity [18], [19].

Cordon-bleus (i.e. species of the genus *Uraeginthus*) rarely produce undirected song in the presence of the mate, but rather when they are separated from the mate, and therefore their undirected song has also been called solitary song [20], [21]. Thus, in cordon-bleus, solitary song is easily amenable to bioacoustic research as it can be reliably induced under controlled conditions by separating members of a pair from each other.

In many passerine species the song of females is less complex and/or shorter than in males as for example in red-cheeked cordon-bleus (*Uraeginthus bengalus*), a congener of our study species [20] or in black-bellied wrens (*Thryothorus fasciatoventris*) [22]. Song structure can also be just slightly different between females and males as in European starlings (*Sturnus vulgaris* [23], but see [24]) or indistinguishable between sexes, as for instance in the forest weaver (*Ploceus bicolor* [25]).

The song of passerine birds is generally believed to be under the control of gonadal hormones affecting song control nuclei in the brain by binding to hormone receptors (e.g. [26]). In zebra finches, castration of adult males reduced the amount of both directed and undirected song [27], [28]. Although both kinds of song can be restored with testosterone replacement, hormone sensitivity varies; undirected singing has a lower threshold for hormonal activation than directed song [27]. Furthermore, the conversion of androgens to estrogens plays an important role for directed song - but not for undirected song, as inhibiting the transformation of androgen to oestrogen (by aromatase blocker) reduces the likelihood that males produce directed song, while undirected song remains unaffected [29].

The endocrine control of female song is not well understood. Studies on European starlings [30], [31] and European robins (*Erithacus rubecula*, [32]) suggest that like in males, testosterone plays an important role for song production in females. In addition, in many species in which females do not normally produce song or sing rarely, they can be induced to sing by treating them with testosterone, (e.g. [30], [31], [33]–[37]). In the zebra finch, however, adult females cannot be induced to sing by testosterone treatment unless they received testosterone or estradiol treatment as hatchlings. Thus, early estradiol action seems to masculinise the female song system and enables the induction of singing behaviour by testosterone administration (e.g. [38]–[40]). Females masculinised as such may even sing spontaneously without further treatment when adult [41].

Solitary (or undirected) song has never been compared between females and males of a species in which both sexes regularly sing. Also, it is unclear whether the occurrence of solitary song in such a species correlates with an increase in testosterone in both sexes. To answer these questions, we induced members of a pair of blue-capped cordon-bleus to produce solitary song by separating them from each other and took blood samples to determine plasma testosterone levels. We compared the solitary song of females and males by conducting a detailed multi-parametric analysis of their song. Our aims were to (1) describe the solitary song in blue-capped cordon-bleus and to (2) compare it to published descriptions of the song of zebra finches. Furthermore, we wanted to (3) test for sex-specific differences in solitary song and (4) to investigate whether occurrence, characteristics and performance of solitary song correlates with the level of testosterone.

## Methods

### Subjects and housing

The study was conducted on eighteen adult blue-capped cordon-bleus (nine of each sex) that formed nine pairs which had at least produced fertile eggs together. Fourteen subjects (8 females, 6 males) were purchased from six different commercial breeders or pet shops and four subjects (1 female, 3 males) were reared by two different of our breeding pairs. Throughout the study, subjects were kept under non-breeding conditions, i.e. they did not have access to nesting material and were fed with non-breeding diet (*ad libitum*). Non-breeding diet consists of drinking water, cuttlebone, bird's grit and a tropical seed mixture. This was supplemented once a week with cucumber and a special food mixture which contained frosted and dried insects, egg food, dried herbs, grated carrots and apples, minerals (Supramin), micronutrients (Nektron) and vitamins (Quikon Forte).

For the study, birds were housed as pairs in sound-isolation chambers measuring 70 cm×50 cm and 50 cm high from inside and containing a metal wire cage equipped with three wooden perches (home cages). Birds were kept on a 14∶10 light∶dark schedule (lights on 07:00–21:00 Central European time) at ca. 22°C and ca. 50% humidity.

### Experimental procedure and sampling of blood plasma

To induce solitary song (solitary-song-induced condition), the male and the female of a given pair were moved to two different sound-isolation chambers containing a cage equipped identically as the home cage (standard equipped). In the baseline condition, both members of a pair were moved together to a sound-isolation chamber containing a standard equipped cage that was not their home cage, to control for the potential confounding effect of being caught and moved into another environment. Relocation of subjects took place between 10:00 and 11:00 hours and lasted for one hour. Sixty minutes later (60±2.7 min), blood samples were taken of both subjects simultaneously by wing vein puncture with a 19-gauge hypodermic needle and by collecting the blood in heparinised micro-hematocrit tubes. Afterwards, subjects were transferred into their home cages. Subsequently, blood plasma was obtained by centrifugation of whole blood and stored at −80°C upon analysis. Experiments were repeated two weeks later if (1) not enough blood plasma could be collected for the hormone assay in the first trial (11 cases), (2) no solitary song was induced in the solitary-song-induced condition (2 cases) or (3) subjects produced song in the baseline condition (1 case). Five pairs received the solitary-song-induced treatment first and four pairs received the baseline treatment first. Where experiments had to be repeated the two types of treatments were alternated. If samples of blood plasma of a given subject obtained during the first trial were too small for hormone analysis, samples from the same subject obtained during repeated trials conducted under the same condition (either baseline or solitary-song-induced) were pooled for analysis. Hormone levels revealed for such pooled samples did not differ from those of single samples (baseline condition: Mann–Whitney U test, U = 14, n1 = 13, n2 = 4, p = 0.20; solitary-induced condition: independent t-test, t = 0.54; df = 16; p = 0.60). Procedures were in accordance with national laws and approved by the Government of Upper Bavaria according to the Tierschutzgesetz (approval number 55.2-1-54-2531.3-59-07).

### Hormone assays

Testosterone concentration was determined by direct radioimmunoassay (RIA), following [42]. Mean±SD extraction efficiency for plasma testosterone was 86.3±0.1%. All samples were analysed in one assay. Standard curves and sample concentrations were calculated with Immunofit 3.0 (Beckman Inc. Fullerton, CA), using a four parameter logistic curve fit. The lower detection limit of the standard curve was determined as the first value outside the 95% confidence intervals for the zero standard (B_max_) and was 0.41 pg/tube. The intra-assay coefficient of variation was 6.8%. Because the testosterone antibody used shows significant cross-reactions with 5a-dihydrotestosterone (44%) our measurement may include a fraction of 5a-DHT. From the baseline measures of the males, one outlier (>2 SD of the mean) was excluded from the analyses (sample sizes are reduced in the relevant subset of data).

### Sound recordings

In each sound-isolation chamber a microphone (Earthworks TC20) was mounted on the ceiling and with a ca. 35–45° angle from the ceiling pointing to the centre of the cage. The microphone was connected to a PR8E amplifier (SM Pro Audio), which feed into an Edirol USB audio capture device (Edirol UA 1000) connected to a computer. Sounds were recorded at a sampling rate of 44 kHz and 16 bit resolution using Sound Analysis Pro version 2.062 (SAP; [43]; freely available at: http://ofer.sci.ccny.cuny.edu/html/sound_analysis.html).

### Sound analysis

We considered the first fifty songs (mean ± SD mean: 50.8±13) a subject produced after separation from the mate (when no song was produced in the first trial we selected songs of subsequent trials). We ignored song renditions that were preliminarily aborted by the subject and thus not performed in its full length.

**Figure 1:**
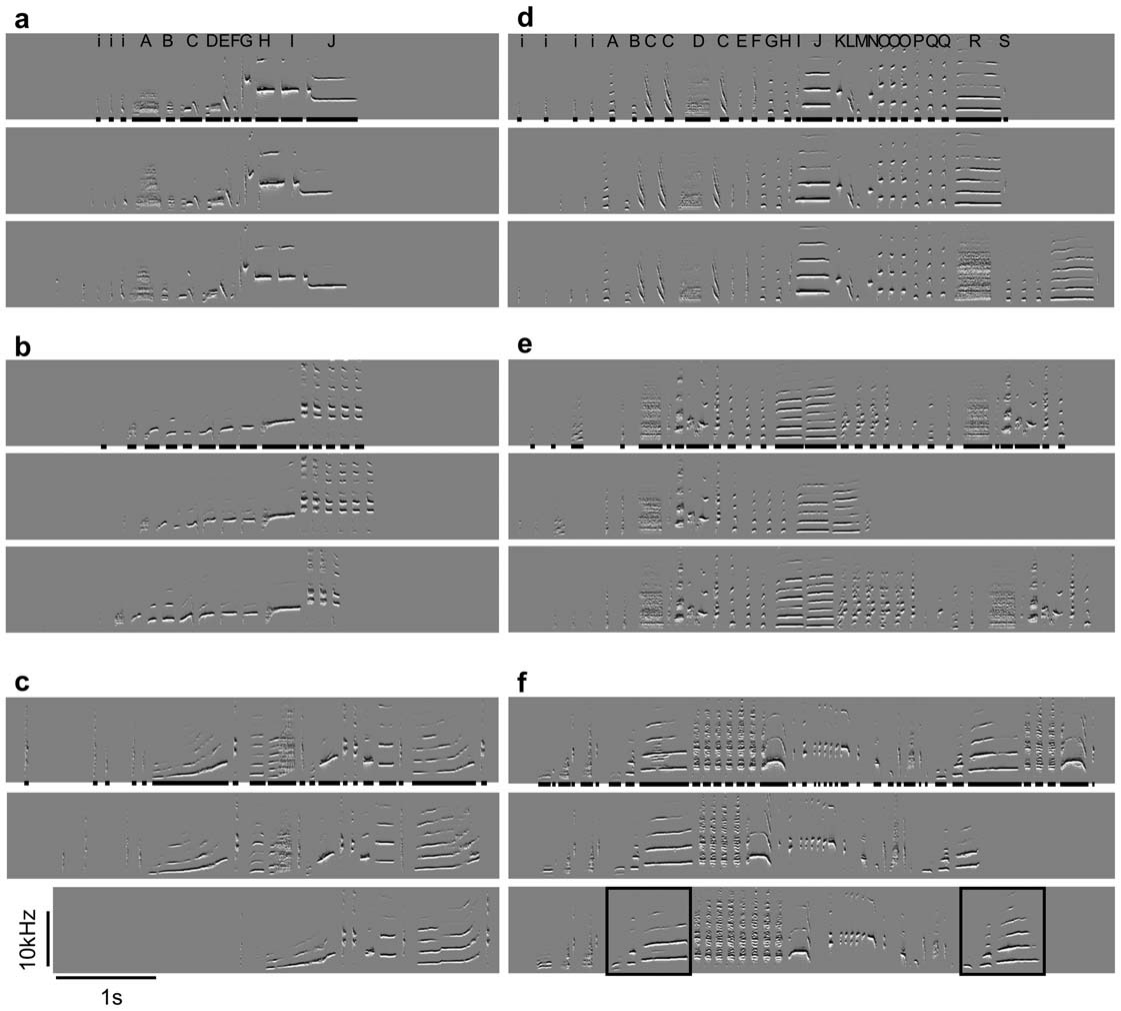
Solitary song of female and male blue-capped cordon-bleus. Each subject sang an individually characteristic song consisting of a unique sequence of syllables with some rendition to rendition variation. Sexual dimorphism in the song was subtle (cf. Table 2). Shown are images of spectral derivatives of solitary song (frequency as a function of time). The left panel (a–c) gives three examples of song renditions for each of three female subjects; the right panel (d–f) gives three examples of song renditions for each of three male subjects. Black bars on the bottom of each top image indicate syllables delineated using a 23 db threshold for amplitude and a −2.1 threshold for entropy in the features batch window of SAP. For the top images of (a) and (d), syllable labels are indicated as follows, i: introductory syllable, A, B, C etc. subsequently occurring syllable types. Note the immediately repeated syllables C, O and Q in (d). In the third rendition from the top of (f) a motif consisting of three syllables that are repeated within the same song is highlighted by a black box. See supplementary material for sound files for (a) and (d).

In a first step, we extracted several acoustic parameters using SAP. To this end, syllables were automatically delineated using a constant threshold for amplitude (23 dB) and entropy (−2.1) in the features batch window (Fig. 1). These thresholds turned out to be most appropriate for this species after comparing delineation with different settings.

In a next step, we printed images of spectral derivatives (representing the change of power rather than power itself, [44]) using SAP. Based on these images and the automatic delineation of syllables we visually compared song renditions of a given subject and labelled the same syllables with the same letter according to their overall spectro-temporal appearance. This allowed us to determine the repertoire size in terms of the number of song types as well as the number of syllable types for each subject. Blue-capped cordon-bleus usually initiate a song with a variable number of introductory syllables, which were labelled as introductory syllables and not further categorized. We excluded introductory syllables from all further analysis.

Cumulative curves of new syllable types plotted against the number of syllables analysed were visually checked (e.g. [45], [46]). They all reached saturation, indicating that the number of songs sampled was sufficient to cover the syllable repertoire in this species.

The features batch procedure in SAP provided us with parameter tables for all syllables and we manually added a column with the syllable label resulting from visual inspection.

From these parameter tables and the visual inspection of images we extracted six spectro-temporal features (duration, mean pitch, frequency modulation, entropy, pitch goodness, mean frequency, [44]) and further overall and derived song features (see Table 1). For a given syllable type of a subject we averaged spectro-temporal features of all renditions of that syllable type (excluding introductory syllables). We compared differences in those features between sexes by entering values for all syllable types that a female or male subject produced into the analysis. As such values might be confounded by sex-specific differences in the repertoire composition (e.g. males could have more harmonic structures in their repertoires and therefore reach higher values of pitch goodness), we compared the repertoire compositions between females and males. As examples, we chose two characteristic classes of syllables, buzz syllables (rapid and repeated frequency modulation, with few obvious harmonics) and flat syllables (syllables containing harmonic structures also termed ‘harmonic stacks’, cf. [47], [48]) and compared their proportion in the repertoires between sexes. To this end, we visually classified all types of syllables as either buzz syllables, harmonic stacks or remaining syllables following definitions of Leadbeater et al. [47] and Sturdy et al. [48].

**Table 1:** List and description of song features.

Song features	Description
**Overall song features**	
size of song type repertoire	number of different song types in the repertoire of a subject
size of syllable type repertoire	number of different syllable types in the repertoire of a subject
% repeated syllables	number of syllables that occur two or more times in a row divided by the total number of syllables
song duration (ms)	song duration measured from the onset of the first syllable after the introductory syllables to the offset of the last syllable as delineated in SAP
number of syllables per song	number of syllables per song not taken into account introductory syllables
tempo (s^−1^)	number of syllables per seconds within a song
increment of pitch (Hz/ms)	difference in pitch from first to last syllable within a song divided by song duration
correlation pitch/sequential order	correlation coefficient between mean pitch of a syllable and its sequential order in the song, describes the change of pitch
**Spectro-temporal features (SAP)**	
syllable duration (ms)	time elapsing from the onset to the offset of a syllable
mean pitch (Hz)	for harmonic sounds: fundamental frequency; otherwise: mean frequency
mean frequency modulation	estimate of absolute slope of frequency traces
mean entropy	width and uniformity of power spectrum, measure of noisiness
mean pitch goodness	periodicity or harmonicity of the sound
mean mean frequency (Hz)	estimate of the centre of derivative power
**Spectro-temporal stereotypy**	
min cv syllable duration	minimum coefficient of variation of duration
min cv mean pitch	minimum coefficient of variation of pitch
min cv mean frequency modulation	minimum coefficient of variation of frequency modulation
min cv mean entropy	minimum coefficient of variation of entropy
min cv mean pitch goodness	minimum coefficient of variation of pitch goodness
min cv mean mean frequency	minimum coefficient of variation of mean frequency
**Sequential stereotypy**	
internal linearity score	sequential order of syllables in the song (see text)
consistency score	probability of consistent syllable transitions (see text)
**Performance related song features**	
number of songs	total number of songs in 1 h of separation (first trial)
latency to sing (s)	latency to sing after separation from mate (first trial)

When visually inspecting the images it seemed that subjects increase the pitch of their syllables throughout the song. To verify this observation we compared the pitch of the first and the last syllable of a given song. We were also interested whether there was a sex-specific difference in this increase and to test this we calculated the increment of pitch by dividing the difference in pitch from first to last syllable by the given song duration and we calculated the correlation coefficients between pitch and sequential order of a given syllable. As performance related song features we assessed the latency to sing in seconds from the start of separation from the mate by subtracting the time of the onset of the first song from the start of the experiment. We here only considered the first trial except for two females that did not sing solitary song during the first trial for which we took the latency of the second trial. In addition, we counted the number of songs produced during the one hour of separation (Table 1).

### Measuring song stereotypy

We measured the song stereotypy on two different levels: (1) spectro-temporal stereotypy of syllables and (2) stereotypy of syllable sequences. To estimate spectro-temporal stereotypy we calculated the coefficient of variation (cv = SD/mean) of the spectro-temporal features provided by SAP. For reasons of comparability, we only considered the four most frequent syllables of each subject because the smallest syllable repertoire observed contained four syllables. For those syllables we selected the first 20 renditions produced after separation from the mate. For between-subjects comparisons, we choose the cv for the syllable with the lowest cv for each subject, i.e. the syllable with the lowest variability from rendition to rendition (minimum cv per subject).

To estimate sequential stereotypy we calculated internal linearity and consistency following the approach described by Scharff and Nottebohm [49] and modified e.g. by Bottjer and Altenau [50]. These calculations were based on the sequences of syllable labels resulted from visual inspection of images (see above). Briefly, internal linearity was calculated as the number of different syllables produced divided by the number of different syllable-to-syllable transitions (excluding introductory syllables and transitions at the ends of songs). A song with a fixed sequence of syllables (e.g. always A-B-C-D) is perfectly linear and receives a score of 1, whereas a score close to zero indicates high variability in the sequential order from rendition to rendition. Consistency was calculated as the number of the most frequent transition for each syllable by the total number of transitions for this syllable. Complete consistency is represented by a score of 1, while songs with smaller scores are less consistent.

### Statistical analysis

Statistical tests were conducted using SPSS 15.0 and R version 2.13.0 (http://www.r-project.org/). Where several measurements per subject were available we used linear mixed-effects models implemented in R2.13.0 with the add-on package nlme and entered identity of subjects as random factor. Where only one value per subject was available for a given song feature (e.g. size of repertoire) we used independent samples t-test for equality of means when testing for differences between sexes if data were normally distributed or otherwise Mann-Whitney U tests. We used Shapiro-Wilk-tests to check for normality of data. We used a paired-samples t-test when comparing the pitch of the first to last syllables (those data reached criteria for parametric testing). When testing for correlations between acoustic and hormonal data we used Pearson's correlations tests when data where distributed normally and Spearman's rank correlations otherwise. All tests were two-tailed and we calculated exact p values, when samples were too small for asymptotic variants of nonparametric tests [51]–[53]. To control for multiple testing when comparing several song features between females and males we applied the Benjamini-Hochberg false discovery rate procedure within the different categories of song features and provide adjusted P values accordingly [54].

## Results

### Description of blue-capped cordon-bleu song

**Figure 2:**
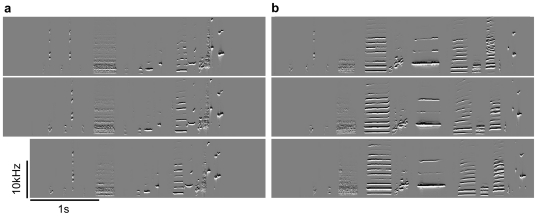
Different song types of one male blue-capped cordon-bleu. A few blue-capped cordon-bleus sang more than one song type each consisting of a unique sequence of syllables. Shown are images of spectral derivatives of solitary song (frequency as a function of time). (a) and (b) give three examples of song renditions for each of two song types sung by one and the same male.

Blue-capped cordon-bleu song is initiated by introductory syllables, contains harmonic stacks and a given bird typically sings only one song type which is characterised by a unique sequence of syllables (Fig. 1, Sound file S1 and S2). However, there can be substantial intra-individual variation from rendition to rendition of a song. For instance, a part of the typical sequence can be missing in some renditions (cf. third rendition from top in Fig. 1c and first and second rendition from top in Fig. 1d) or single syllables can be repeated a different number of times (cf. Fig. 1e). Some of the subjects produced different song types (up to 4 in the current data set) each characterised by a unique sequence of syllables (Fig. 2, Table 2).

**Table 2:** Song features in female and male blue-capped cordon-bleus.

	Females (n = 9)	Males (n = 9)				
	mean±SD	mean±SD	T	df	P	P adjusted
**Overall song features**						
size of song type repertoire^a^	1±0	1.56±1.01	27*		0.21	0.41
size of syllable type repertoire^a^	11.89±5.3	24.67±7.16	−4.30	16	**0.001**	**0.004**
% repeated syllables^b^	15.2±19.96	17.7±12.32	−0.39	16	0.70	0.83
song duration (ms)^b^	2060.26±559.67	3026.7±1078.69	−2.44	16	**0.03**	0.08
number of syllables per song^b^	10.72±3.64	16.19±6.39	−2.40	16	**0.03**	0.08
tempo (s^−1^)^b^	5.14±1.46	5.32±1.24	−0.36	16	0.73	0.83
increment of pitch (Hz/ms)^b^	0.97±0.59	0.94±0.51	0.08	16	0.94	0.94
correlation pitch/sequential order^b^	0.40±0.27	0.46±0.21	−0.53	16	0.60	0.83
**Spectro-temporal features**						
syllable duration (ms)^b^	161.75±62.29	149.22±26.77	0.25	16	0.81	0.81
mean pitch (Hz)^b^	3088.56±589.08	2564.78±640.34	2.00	16	0.06	0.24
mean frequency modulation^b^	27.12±5.48	29.15±6.27	−0.58	16	0.57	0.70
mean entropy^b^	−4.42±0.45	−3.89±0.69	−1.87	16	0.08	0.24
mean pitch goodness^b^	153.60±34.57	173.08±44.41	−1.12	16	0.28	0.56
mean mean frequency (Hz)^b^	4343.13±590.67	4136.77±310.50	0.57	16	0.58	0.70
**Measures of stereotypy**						
**Spectro-temporal stereotypy (min coefficient of variation of the relevant feature)**						
syllable duration^a^	0.08±0.03	0.06±0.03	1.18	16	0.25	0.46
mean pitch^a^	0.07±0.03	0.09±0.04	30*		0.39	0.46
mean frequency modulation^a^	0.09±0.02	0.08±0.02	1.36	16	0.19	0.46
mean entropy^a^	0.14±0.08	0.13±0.06	0.38	16	0.71	0.71
mean pitch goodness^a^	0.12±0.04	0.07±0.02	5*		**0.001**	**0.005**
mean mean frequency^a^	0.03±0.02	0.04±0.02	−0.95	16	0.36	0.46
**Sequential stereotypy**						
internal linearity score^a^	0.61±0.26	0.64±0.18	−0.24	16	0.81	0.81
consistency score^a^	0.86±0.14	0.9±0.07	−0.76	16	0.46	0.81
**Performance related song features**						
number of songs^a^	71±66.43	151.11±112.7	−1.84	16	0.08	0.08
latency to sing (s)^a^	1005.67±706.15	351.22±284.69	2.58	16	**0.02**	**0.04**

a features with single values per subject analysed by independent t-test or

*Mann-Whitney U in cases where data were not normally distributed.

b features with multiple values per subject analysed by linear mixed-effects models. Significant p values are given in bold.

Many subjects immediately repeated some of their syllables within a given song (5/9 females and 8/9 males, cf. Fig. 1). In addition, some subjects repeated stereotyped sequences of two or more syllables (i.e. motifs) within a given song (3/9 females and 2/9 males, cf. third rendition from top in Fig. 1f). In blue-capped cordon-bleus, pitch of syllables increased from the first to the last syllable and this difference was significant both in females (paired-samples t-test: t = −5.64, df = 8, p = 0.0005, pitch of first syllable = 2239±624 Hz, pitch of last syllable = 4053±1095 Hz) and in males (paired-samples t-test: t = −7.79, df = 8, p = 0.0001, pitch of first syllable = 1312±379 Hz, pitch of last syllable = 3609±910 Hz). Note that the first syllable was, however, not necessarily the lowest-pitch syllable and the last syllable not necessarily the highest-pitched syllable in the song (cf. Table 1).

### Comparison of song features between female and male blue-capped cordon-bleus

**Figure 3:**
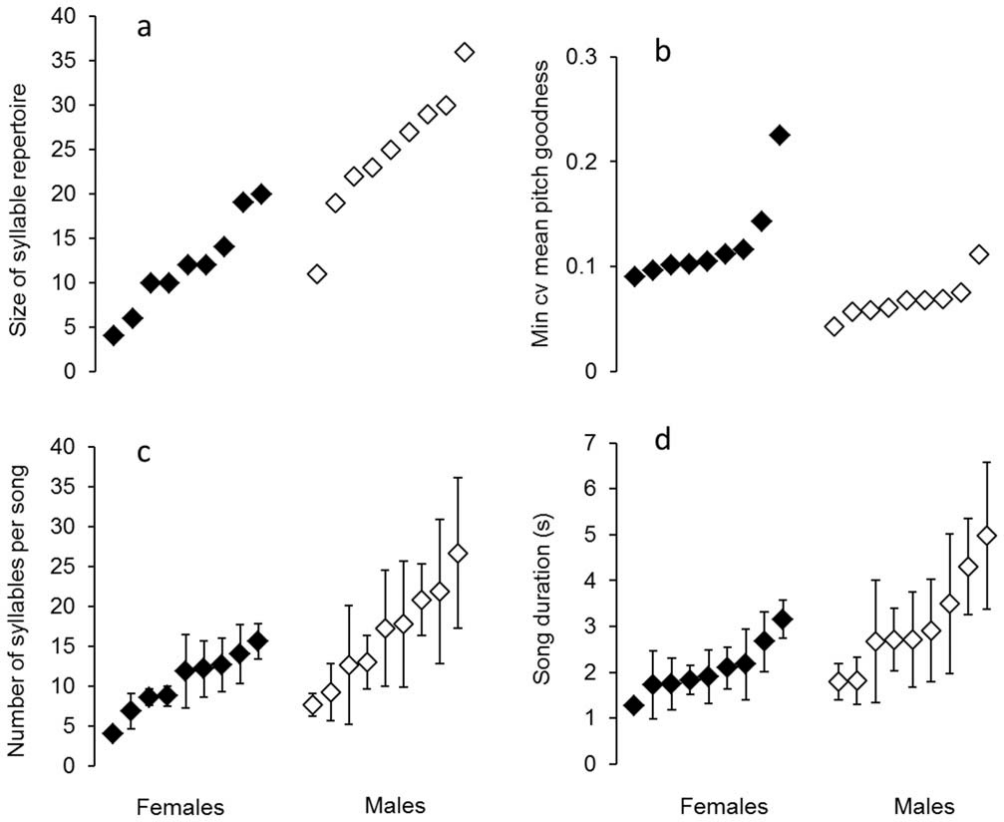
Song features that differed between female and male blue-capped cordon-bleus. (a) size of syllable repertoire, (b) minimum coefficient of variation for mean pitch goodness, (c) number of syllables per song, (d) duration of song (s). For (a) and (b) single values per subject, for (c) and (d) individual means ± SD are displayed. Note that for each feature subjects are displayed in ascending order within their sex group. Filled symbols represent females, open symbols represent males. For statistics see Table 2.

Males sang twice as many different syllable types than females (25 versus 12, see Table 2, Fig. 3). Also, their song tended to be longer than that of females and this seemed to be due to the larger number of syllables that males incorporated into a given song - even though the difference in the number of syllables per song between females and males was not significant after correction for multiple testing (Table 2, Fig. 3). Females and males did not differ in the size of song type repertoires, the proportion of repeated syllables, the tempo and the change of pitch, both measured as the difference from last to first syllable and as correlation between pitch and sequential order of a syllable. There were no clear differences in the spectro-temporal features of females and males, i.e. none of these traits revealed a significant difference after correction for multiple testing (Table 2). However, males reached a higher level of song stereotypy with regard to pitch goodness: for this parameter males reached a significantly lower rendition-to-rendition variation than females (Fig. 3). All other measures of spectro-temporal stereotypy failed to detect sex-specific differences in the song of blue-capped cordon-bleus, and females and males did also not differ with regard to the sequential stereotypy (Table 2). The sexual dimorphism in song stereotypy with regard to pitch goodness might be related to a higher abundance of harmonic stacks in the song of males. To rule out this possibility, we compared the proportions of harmonic stacks and another characteristic class of syllables, the buzz syllables (rapid and repeated frequency modulation, with few obvious harmonics) in the repertoires of females and males. Harmonic stacks made up 24.5±23.5% of syllables in the repertoires of females and 29.2±10.4% in the repertoires of males and this proportion was not different (independent samples t-test: t = −0.55, df = 16, p = 0.59). Buzz syllables made up 13.7±1.3% of syllables in the repertoires of females and 11.2±0.3% in the repertoires of males and this proportion was also not different (independent samples t-test: t = 0.56, df = 9, p = 0.59).

In addition, we measured two performance related song traits and compared them between the sexes: males tended to sing more songs during one hour of separation from the mate and they started singing earlier after their mate had been removed (Table 2).

### Endocrine correlates of solitary song

**Figure 4:**
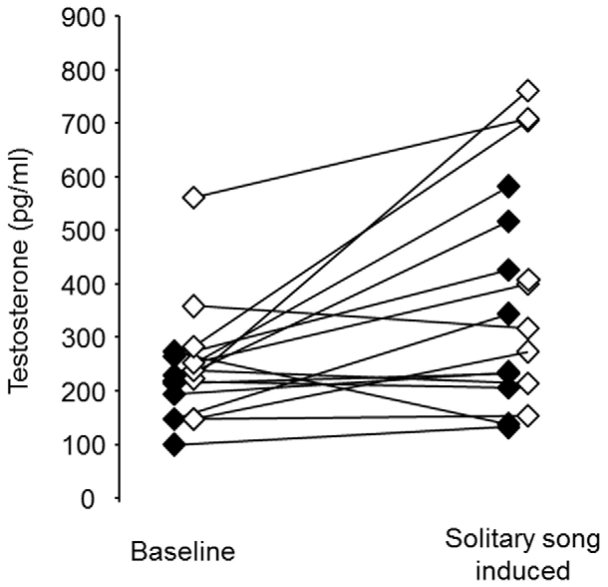
Testosterone was higher in the solitary-song-induced condition compared to the baseline condition. Testosterone concentration (pg/ml) in the blood plasma collected after one hour of baseline treatment (i.e. being transferred into another sound-isolation chamber together with the mate) and one hour of solitary-song-induced treatment (i.e. being transferred into another sound-isolation chamber without the mate). Filled symbols represent females, open symbols represent males.

**Figure 5:**
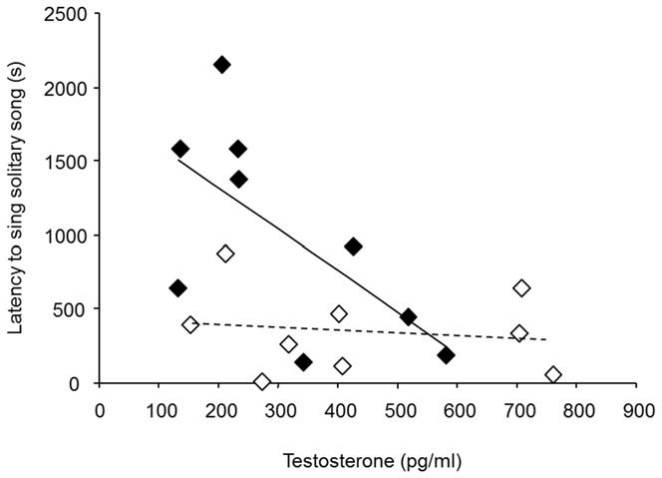
All males and females with high testosterone levels sang rapidly after separation from their mate. Latency to sing solitary song (s) as a function of testosterone concentration (pg/ml) in the blood plasma. Filled symbols and the solid line represent females; open symbols and the broken line represent males.

To investigate whether the occurrence of solitary song was correlated with an increase of testosterone, we first compared the level of testosterone during baseline condition and during solitary-song-induced condition. As there were no differences in the level of testosterone between males and females in both conditions (baseline: independent samples t-test: t = −1.44, df = 15, p = 0.17; solitary-song-induced: independent samples t-test: t = −1.33, df = 16, p = 0.20) we pooled data of males and females to compare between the different treatment conditions. Birds had significantly higher levels of testosterone in the solitary-induced condition than in the baseline condition (mean ± SD mean: 375.29±204.70 versus 239.32±103.20 pg/ml, paired-samples t-test: t = −3.05, df = 16, p = 0.008, Fig. 4) and when testing the sexes separately a trend remained for both of them (paired-samples t-test: females t = −2.02, df = 8, p = 0.08; males: t = −2.22, df = 7, p = 0.06, Fig. 4). Reanalysing these data including the one outlier (one male had a level of testosterone of 1767.84 pg/ml in the baseline condition) did not change the results except that males' levels of testosterone in the reanalysis clearly did not differ between baseline and solitary-induced condition when tested separately. Furthermore, we compared levels of testosterone in the solitary-song-induced condition with performance related song features. Testosterone did not correlate with the number of songs produced in the one hour of separation (Pearson's correlation test: r = 0.27, N = 18, p = 0.28). Visual inspection of the graphs plotting latency to sing against the concentration of testosterone suggested a trend in females, but the correlation just failed to be significant (Pearson's correlation test: r = −0.65, N = 9, p = 0.06; Fig. 5). In males, no such trend could be observed (Pearson's correlation test: r = −0.15, N = 9, p = 0.69; Fig. 5). Thus, those blue-capped cordon-bleu females with higher levels of testosterone tended to sing earlier after their mate was removed, besides that all males started to sing early. However, when analysing data of females and males together, the relationship between testosterone and latency to sing becomes significant (Pearson's correlation test: r = −0.49, N = 18, p = 0.04). Thus, further studies are needed to confirm that this is an effect in females only. Finally, we compared levels of testosterone in the solitary-song-induced condition (pooled for both sexes) with overall song features, spectro-temporal features and stereotypy. None of these parameters correlated with the level of testosterone (Spearman's rank or Pearson's correlations test: overall song features: |r|<0.32, N = 18, p>0.19; spectro-temporal features: |r|<0.37, N = 18, p>0.12; spectro-temporal stereotypy: |r|<0.25, N = 18, p>0.30, sequential stereotypy: |r|<0.18, N = 18, p>0.47).

## Discussion

### Comparison with zebra finch song

Zebra finch song consists of a series of introductory syllables followed by two to four repetitions of an individually distinct motif, which is a stereotyped sequence of several (usually four to seven) distinctive syllables. Songs are usually produced in bouts with each male singing an individually distinct song [16], [55]. A characteristic feature of zebra finch syllables is that they often contain multi-component harmonic structures. Infrequently, zebra finches immediately repeat a given syllable within a motif, such immediate repetition has only been described for captive birds and amongst them only in a small fraction (7%, [56]). The duration of undirected song in zebra finches is on average 5170 ms [57]. Mean pitch of undirected song lies between 983 and 1092 Hz. Pitch goodness, a measure for how much energy lies in harmonics, reaches levels from 35 to 38. Mean values of frequency modulation are 28 to 31 and entropy, a measure for noisiness, adopts values between −2.9 to −2.5 (all measured for undirected song, [58]). Finally, measures for sequential stereotypy reveal an internal linearity score of 0.95 and a consistency score of 0.96 for zebra finch undirected song [50].

Blue-capped cordon-bleus belong to the same family (Estrildidae) as the zebra finch. It is therefore not surprising that they share some song features with zebra finches: for instance, in both species, song is mainly produced in two different social contexts, and a given bird normally sings only one individually distinct song type. Another typical feature of both species is that the song contains multi-component harmonic structures. Like zebra finches, blue-capped cordon-bleus show the phenomenon of immediate syllable repetition within a given song, however, such repetition occurred much more often in our subjects than in zebra finches.

Most of the spectro-temporal features vary between the two species: blue-capped cordon-bleus sang at higher pitch and their songs were shorter than those of zebra finches. Their song reached higher levels of pitch goodness than that of zebra finches, i.e. contained a higher proportion of energy in multi-component harmonic structures. Frequency modulation was similar in both species but entropy was much higher in zebra finches than in blue-capped cordon-bleus, indicating that the song of blue-capped cordon-bleus is less noisy than the song of zebra finches.

In the majority of blue-capped cordon-bleus (72%) the song was not organised in motifs, that is, birds produced the sequence of their syllables only once within a song. In this respect, song organisation varies from that of zebra finches, which repeat stereotyped sequences of syllables (i.e. motifs) several times within a song. Overall, the sequential order of syllables was more variable in blue-capped cordon-bleus than in zebra finches and they were also less consistent in following particular sequences. The most striking difference between the species is that in zebra finches only males sing, whereas in blue-capped cordon-bleus both females and males sing – the reason for us to establish them as a new model species.

### Comparison of female and male song in blue-capped cordon-bleus

Syllable repertoire size was twice as large in males and thus males reached a higher level of song diversity than females. Also the song of males tended to be longer in duration. This is in line with findings on some but not all other studied species, in which females regularly sing. In female black-bellied wrens for instance, songs were shorter and their repertoires smaller than in males [22] and in red-cheeked cordon-bleus, songs of females were shorter than that of males [20]. Likewise, zebra finch females that had received an estradiol-treatment as hatchlings to masculinise their song system produced shorter songs than males as adults [39]. In other, mostly duetting, species both sexes have been reported to reach the same level of song diversity (e.g. bay wren, *Thryothorus nigricapillus*, [59]; forest weaver, [60]; white-browed sparrow weavers, *Plocepasser mahali*, [61]). The diversity of female relative to male song forms a continuum between species with male-only song production on one end and species with equality of song diversity on the other [62]. Blue-capped cordon-bleus seem to be intermediate between these two extremes.

In addition to song diversity, we found another clear sexual dimorphism in the rendition-to-rendition variation of pitch goodness: males reached a lower level of variation in the pureness of multi-component harmonic structures. Thus, their song was more stereotyped with respect to this parameter and since males did not have more harmonic stacks in their repertoires than females, this difference was not due to differences in the repertoire composition. All the other measurers of stereotypy did not differ between females and males. Thus, in comparison to other species, in which females clearly show a higher level of song variability (e.g. canaries, *Serinus canaria*, [63], Northern cardinal, *Cardinalis cardinalis*, [64]) differences in song stereotypy in blue-capped cordon-bleus seem rather subtle. Sexes did, however, vary in performance related song features: males tended to sing more songs and started to sing earlier during the one hour of separation. Besides these dimorphisms in syllable repertoire, song duration, pitch goodness and the performance related features, all of the remaining song features did not differ significantly between the sexes.

In the current study we did not compare song features between members of a pair-bond. Blue-capped cordon-bleus would, however, provide a perfect model to investigate whether vocal convergence occurs between partners such as has been described for instance in budgerigars (*Melopsittacus undulates*, [65]).

### Endocrine correlates of solitary song

The overall plasma testosterone concentrations of blue-capped cordon-bleus were similar in both sexes. This is in contrast to findings in zebra finches were males have higher levels of testosterone (mean ± SD mean: 1434±126 pg/ml, Table 5 in [66]) than females (mean ± SD mean: 400±438 pg/ml, Fig. 1 in [67]) and even in Northern cardinals, another species with female and male song production, males had slightly higher levels of testosterone than females (mean ± SE: 1832±130 pg/ml versus mean ± SE: 1440±38 pg/ml, [68]). In blue-capped cordon-bleus, the fact that both females and males sing is paralleled by the lack of a sexual dimorphism in testosterone profiles. Testosterone levels in cordon-bleus did also not correlate with the subtle sexual dimorphic song motor pattern. Thus, neither any overall song feature (such as repertoire size, song duration etc.) nor any spectro-temporal feature or song stereotypy correlated with the level of testosterone. Also the number of songs produced did not correlate with testosterone, and this is in contrast to findings on male zebra finches, in which such a correlation was demonstrated [66].

Although testosterone levels do not explain sex differences in song pattern, they might be related to singing activity. Despite the relatively low levels of testosterone in blue-capped cordon-bleus, solitary song occurred in parallel to an increase in testosterone. The correlative nature of this finding does not allow us to rule out the possibility that a third factor (e.g. being stressed by the absence of the mate) could lead to higher levels of testosterone on one hand and to higher song output on the other. The finding that those females that sang earlier tended to have higher testosterone levels, suggests a causal relationship between the hormone level and solitary song at least in females, although given that this correlation becomes significant only if females and males are analysed together, further studies would be needed to confirm that this is an effect in females only. A causal relationship between testosterone and the onset of directed song in male zebra finches has been demonstrated experimentally: males that had been implanted with testosterone started to produce directed song earlier after the introduction of a female than before the implantation [69]. Whilst the authors in [69] measured this effect only seven days after the implantation, in principle, steroid hormones can fluctuate in a rapid time course depending on the social context [70]. Furthermore, hormone treatment can influence vocal behaviour within a few minutes, as has been shown for structural vocal parameters in midshipman fish (*Porichthys notatus*, [71]). Thus, it is conceivable that the described increase in testosterone facilitates singing of solitary song in blue-capped cordon-bleus, i.e. small increases in testosterone levels would have large behavioural consequences. Alternatively, there could be a causal link between hormone and behaviour in the opposite direction. Thus, the increase of singing could lead to an increase in testosterone and therefore females that start to sing earlier would have higher hormone levels. Such a relationship has been demonstrated for female ring doves (*Streptopelia risoria*) in which nest cooing vocalisations stimulate the release of hormones that lead to follicular growth and ovulation (reviewed in [72]). Experiments manipulating the hormone levels will be needed the fully understand the relationship between testosterone and singing in blue-capped cordon-bleus.

Testosterone levels of blue-capped cordon-bleus were low in comparison to male zebra finches [66] but within the range of other tropical passerines. Non-breeding testosterone concentrations of 33 tropical species was (mean ± SD) 421.8±428.3 pg/ml (calculated from testosterone concentrations reviewed in [73]). Thus, blue-capped cordon-bleus fit in the general pattern of tropical species having low testosterone concentrations ([1], but see [74]), a phenomenon that might be associated with tropical life history traits such as year-round territoriality and asynchronous breeding [74], [75] or with an immune system function related to greater pathogen burden in the tropics [76]. On a more proximate level, low levels of testosterone might be related to an increase in target sensitivity e.g. by increasing expression of androgen receptors in relevant brain regions [77], [78].

## Supporting Information

Sound File S1 **Solitary song of a female blue-capped cordon-bleu.** An image of the spectral derivative of this song is shown in the top of Figure 1a.
(WAV)

Sound File S2 **Solitary song of a male blue-capped cordon-bleu.** An image of the spectral derivative of this song is shown in the top of Figure 1d.
(WAV)
